# Synthetic lethality in malignant pleural mesothelioma with PARP1 inhibition

**DOI:** 10.1007/s00280-017-3401-y

**Published:** 2017-07-29

**Authors:** Gayathri Srinivasan, Gurjit Singh Sidhu, Elizabeth A. Williamson, Aruna S. Jaiswal, Nasreen Najmunnisa, Keith Wilcoxen, Dennie Jones, Thomas J. George, Robert Hromas

**Affiliations:** 1 Department of Medicine and the Cancer Center, University of Florida Health, 1600 SW Archer Rd, Gainesville, FL 32610 USA; 2 Tesaro, Waltham, MA 02451 USA

**Keywords:** Mesothelioma, BAP1, PARP1, Homologous recombination, Niraparib, Olaparib

## Abstract

Malignant pleural mesotheliomas (MPM) are most often surgically unresectable, and they respond poorly to current chemotherapy and radiation therapy. Between 23 and 64% of malignant pleural mesothelioma have somatic inactivating mutations in the BAP1 gene. BAP1 is a homologous recombination (HR) DNA repair component found in the BRCA1/BARD1 complex. Similar to BRCA1/2 deficient cancers, mutation in the BAP1 gene leads to a deficient HR pathway and increases the reliance on other DNA repair pathways. We hypothesized that BAP1-mutant MPM would require PARP1 for survival, similar to the BRCA1/2 mutant breast and ovarian cancers. Therefore, we used the clinical PARP1 inhibitors niraparib and olaparib to assess whether they could induce synthetic lethality in MPM. Surprisingly, we found that all MPM cell lines examined, regardless of BAP1 status, were addicted to PARP1-mediated DNA repair for survival. We found that niraparib and olaparib exposure markedly decreased clonal survival in multiple MPM cell lines, with and without BAP1 mutations. This clonal cell death may be due to the extensive replication fork collapse and genomic instability that PARP1 inhibition induces in MPM cells. The requirement of MPM cells for PARP1 suggests that they may generally arise from defects in HR DNA repair. More importantly, these data demonstrate that the PARP1 inhibitors could be effective in the treatment of MPM, for which little effective therapy exists.

**Electronic supplementary material:**

The online version of this article (doi:10.1007/s00280-017-3401-y) contains supplementary material, which is available to authorized users.

## Introduction

Malignant pleural mesothelioma (MPM) is a rare and aggressive tumor primarily caused by inhalation of asbestos. Treatment for MPM is most commonly surgery, with chemotherapy/radiotherapy for residual disease [1, 2]. The prognosis for this disease is dismal, given that complete resection of the tumor is difficult, and the lack of sensitivity to chemotherapy or radiation [3]. In addition, response to newer agents, such as the anti-angiogenic biological therapies and the kinase targeted small molecules, has been disappointing [1, 2].

Interestingly, between 23 and 64% of MPMs have somatic inactivating mutations in the BAP1 (BRCA1-associated protein 1) gene [4–7]. BAP1 is also mutated in a fraction of uveal melanomas, renal cell carcinomas, and cholangiocarcinoma [8]. BAP1 is a member of the BRCA1/BARD1 complex and plays a role in homologous recombination (HR) DNA double strand break (DSB) repair [9, 10]. BAP1 interacts with the BRCA1 RING finger domain, which mediates BRCA1’s E3 ubiquitin ligase activity [11]. BAP1 functions as a deubiquitinase and BRCA1 likely recruits it to sites of DSBs to restore histone ubiquitination to native levels after repair is completed [11].

We hypothesized that BAP1-mutant mesothelioma might be addicted to PARP1-mediated DNA repair pathways, similar to the BRCA1/2-mutant breast and ovarian cancers [12–14]. BAP1-mutant MPM would have defects in the HR DNA repair, and thus may also require PARP1 for proliferation. PARP1 inhibition in BRCA1-deficient cancers results in collapsed replication forks and genomic instability leading to mitotic catastrophe and ultimately cell death [12, 13]. When a cancer arises from defects in one DNA repair pathway, such as HR in the BRCA1/2-mutant malignancies, they become addicted to other DNA repair pathways for survival during replication [12–14]. A well-known example of this is the inhibition of the DNA repair component PARP1 in the BRCA1-deficient cancers result in replication fork collapse and mitotic catastrophe [12–14]. In this situation, PARP1 inhibition stalls replication forks, which cannot be repaired because of the underlying defect in HR in these cancers [12]. Sensitivity to PARP1 inhibition is one biomarker of HR deficiency [12, 14].

Significantly, we discovered that the clinical PARP1 inhibitors, niraparib and olaparib, markedly decreased clonal survival in all MPM cell lines tested, regardless of BAP1 status. This implies that defects in homologous recombination DNA repair are at the origin of most MPM. More importantly, these data suggest a new modality for targeted therapy to be studied in a cancer that desperately needs effective treatment.

## Materials and methods

### Cell culture, drug treatment and survival assay

The BAP1-mutant MPM cell lines, H-Meso01, H2452, H2461, H2731, and the wild type BAP1 MPM cell lines CRL-2081 and H290 were cultured in RPMI 1640 medium supplemented with 10% fetal bovine serum, and 1% penicillin and streptomycin. Niraparib and olaparib were purchased from MedChem Express (Monmouth Junction, NJ). Clonal survival assays were performed as described [14]. All experiments were repeated at least three times in triplicate, and no data points were excluded from statistical analysis.

### Nuclear structure assays and DNA damage foci

Nuclear structural abnormalities (micronuclei and bridging) arising from aberrant chromosomal segregation after replication fork fusion were assessed as we described [14]. Briefly, DMSO control and olaparib- or niraparib-treated cells were grown on coverslips, and the cells were fixed in 100% methanol at −20 °C for 20 min for 72 h after niraparib treatment at 3 uM. The fixed cells were mounted using DAPI-Fluoromount G clear mounting media from SouthernBiotech and analyzed within 24 h with laser confocal scanning microscope (TCS-SP5, Leica Microsystems, Exton, PA) as described [14]. At least six distinct determinations (100–150 nuclei per determination) were performed for each group.

Confocal Immunofluorescence foci assays were performed for γ-H2Ax DNA damage foci analysis as we described [14]. The γ-H2AX (S139) primary antibody was from Millipore (Billerica, MA), and the secondary antibody conjugated with Alexa Fluor dye was from Invitrogen (Waltham, MA). After staining, coverslips were mounted with DAPI-Fluoromount G clear mounting media from SouthernBiotech (Birmingham, AL) and analyzed within 24 h with a laser confocal scanning microscope (TCS-SP5). At least six distinct determinations (100–150 nuclei per determination) were performed for each condition for statistical analysis. Cells with more than five foci were counted as positive. Photomicrographs of each distinct cell population were taken at equal magnifications and equal fluorescence intensities.

### Replication fork restart analysis

Replication fork restart after treatment with niraparib was measured using immunofluorescent detection of BrdU foci after DNA denaturation as described [14]. Briefly, after treatment in 3 μM niraparib for 48 h, cells were released into fresh media containing 10 uM BrdU (BD Biosciences, Franklin Lakes, NJ, USA) for 30 min. After washing, cells on coverslip were fixed and the DNA was denatured using hydrochloric acid for BrdU immunostaining. The coverslips were processed for immunostaining using BrdU-specific antibody (Cell Signaling, Danvers, MA, USA) and then incubated with a secondary antibody conjugated with Alexa Fluor dye from Invitrogen for 1 h. Cells were analyzed as above using the laser scanning confocal microscope at the same magnification and the same fluorescence. At least 500 cells were counted for each condition from at least six distinct slides per condition for statistical analysis.

### Cytogenetic and sister chromatid exchange analysis

Structural aberrations in metaphase chromosomes were scored after solid Giemsa staining as we described [14, 15]. Sister chromatid exchanges were analyzed using modifications from previously described protocols [16, 17]. The detailed modifications are as follows: Cells were treated with either 0.06% DMSO or 3 μM of niraparib for 48 h along with BrdU at a final concentration of 5 μg/mL. Forty-eight hours after the treatment, the cells were washed and replaced with fresh media without the drug or BrdU. To this, 10 μg/mL of KaryoMAX colcemid stock (ThermoFisher Scientific, Waltham, MA, USA) was added to give a final concentration of 0.1 μg/mL and incubated for 1 h in the cell culture incubator. The cells were then washed with fresh media and 2 mL of pre-warmed 75 mM KCl was added to the cells and incubated at 37 °C for 10 min. To the KCl, 5–10 drops of 3:1 methanol/acetic acid fixative was added by gently mixing the plate. This solution was removed, and fresh identical fixative was added and incubated at room temperature for 5 min. The fixation step was repeated twice and the coverslips were air-dried. After drying, coverslips were washed twice with PBS for 5 min each at room temperature. The coverslips were then incubated with Hoechst (ThermoFisher Scientific) at a final concentration of 2 μg/mL for 5 min at room temperature and washed briefly in PBS. The coverslips were then placed in 2× SSC buffer and exposed to UV for 10 min followed by rinsing in 2× SSC buffer. The coverslips were then covered with 4% Giemsa stain (Gibco) for 4 min at room temperature. The stain was then washed off in water and the coverslips were mounted on a slide using Fluoromount G (SouthernBiotech) mounting media. Sister chromatid exchange in each group was counted per metaphase on a fluorescent microscope. At least 20 metaphases were counted per condition, and each experiment was repeated three times.

### Statistical analysis

Microsoft Excel or GraphPad Prism software was used for all statistical analyses. An unpaired student *t* test was performed on all data groups. Results were considered statistically significant when *p* values were <0.05.

## Results

### PARP1 inhibition is lethal in MPM cells

**Fig. 1:**
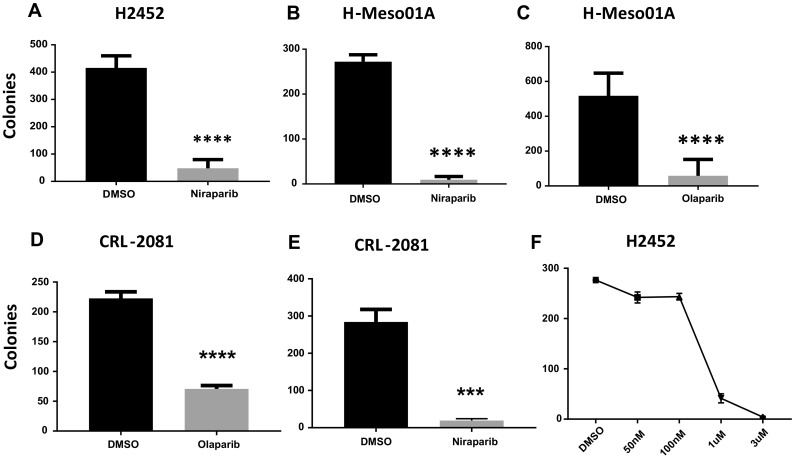
PARP1 inhibition is lethal to MPM cells. Colony formation assays of clonal cell survival with continuous niraparib or olaparib, both at 3 uM. **a** H2452 BPA1-mutant MPM cells exposed to niraparib. **b** HMeso01A BAP1-mutant MPM cells exposed to niraparib. **c** HMeso01A BAP1-mutant MPM cells exposed to olaparib. **d** CRL-2081 BAP1 wild-type MPM cells exposed to olaparib. **e** CRL-2081 BAP1 wild-type MPM cells exposed to niraparib. **f** Dose response of H2452 BPA1-mutant MPM cells exposed to varying concentrations of niraparib. For all figures: * indicates a *p* value of 0.01, ** of 0.001, *** of 0.0001 and **** of < 0.0001

Inhibiting PARP1 in HR-deficient cells, such as the BRCA1- or 2-mutant breast and ovarian cancers, leads to an accumulation of DNA damage from replication fork collapse and ultimately cell death [11, 12]. We tested whether the PARP1 inhibitors niraparib and olaparib could induce clonal cell death in the BAP1-mutant MPM cell lines H2452, H-Meso01A, H2461, H2731, and the BAP1 wild-type MPM cell lines CRL-2081 and H290 [7]. Olaparib and niraparib are both orally active PARP1 inhibitors that are effective in the treatment of ovarian cancers with BRCA1 and BRCA2 mutations [18, 19]. We found that niraparib and olaparib were significantly cytotoxic to each of the MPM cell lines listed above, regardless of the status of BAP1 mutations (Fig. 1 and Supplemental Fig. S1). At the clinically relevant concentration of 3 μM of niraparib, clonal survival of the MPM cell lines averaged <10% (Fig. 1a–d). The BAP1 wild-type MPM cell line CRL-2081 was significantly less sensitive to olaparib than to niraparib (Fig. 1d, e), consistent with the benefit niraparib demonstrated in ovarian cancers that were not deficient in BRCA1 or 2 [19]. A dose response colony formation study in BAP1-mutant MPM cell line H2452 showed that the IC_50_ of niraparib is 400 nM (Fig. 1f).

### PARP1 inhibition in MPM cells leads to replication fork arrest and collapse

**Fig. 2:**
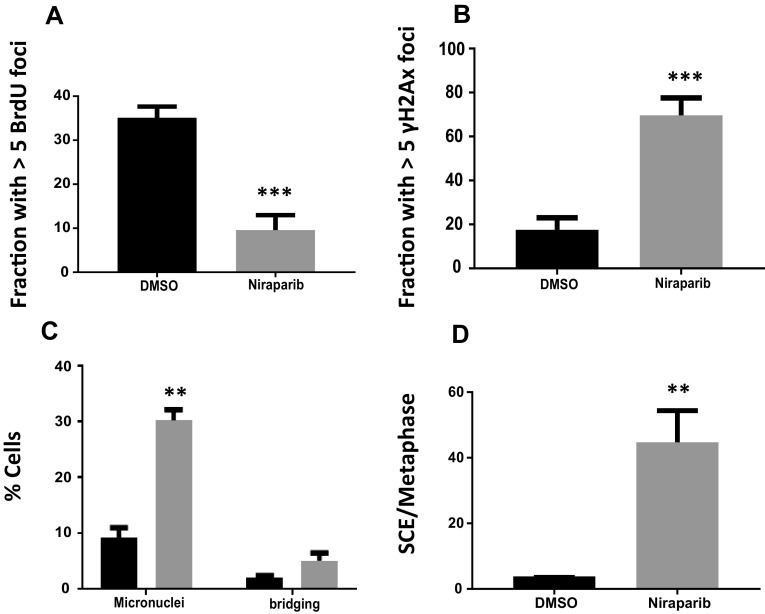
Replication fork and genomic instability in MPM cells after exposure to niraparib. **a** BRDU incorporation assays assessing replication fork repair and restart demonstrating poor fork restart after niraparib exposure in H2452 MPM cells. **b** Confocal immunofluorescence of γ-H2Ax foci demonstrating a marked increase in replication fork structural damage after niraparib exposure in H2452 cells. **c** Genomic instability in BAP1-mutant mesothelioma cells after exposure to niraparib. Confocal immunofluorescence of DAPI-stained H2452 nuclei after exposure to niraparib demonstrated a significant increase in micronuclei and post-mitotic nuclear bridging. **d** Sister chromatid exchange (SCE) assays showing a marked increase in SCE after release from niraparib exposure in H2452 cells

BrdU (Bromo-deoxy uridine) is a thymidine analog that is incorporated into nascent DNA during replication [14]. Active replication forks can be measured by BrdU foci using immunofluorescence. H2542 BAP1-mutant MPM cells treated with 3 μM niraparib for 48 h and then released into fresh media containing BrdU for 30 min showed a 3.5-fold decrease in BrdU foci compared to vehicle control cells (Fig. 2a). This indicated that treating MPM cells with niraparib leads to decreased replication fork restart after removal of niraparib. This implies that the replication forks were damaged beyond repair [12–14].

Stalled replication forks can eventually collapse, which is due to the loss of the replication apparatus at the fork and fork cleavage [12–14]. Collapsed replication forks after replication stress are marked by ATR phosphorylation of H2Ax [14]. These increased γ-H2Ax foci indicate that the replication fork has endured severe structural damage [20]. We found that niraparib treatment of H2452 cells led to a sevenfold increase in the accumulation of γ-H2Ax foci (Fig. 2b, supplemental Fig. S2). The increase in damaged replication forks after niraparib exposure in the MPM cells corresponds with their decreased clonal survival after niraparib.

### PARP1 inhibition results in genomic instability in MPM cells

Replication fork collapse leads to chromosomal fusion events that result in mis-segregated chromosomes during mitosis [12, 14, 15]. Fused chromosomes can have two centromeres, which would result in nuclear bridging, or no centromeres, which result in the chromosome being retained as micronuclei [20]. The presence of bridging or micronuclei indicates genomic instability and mitotic catastrophe [14, 20]. Such bridging and micronuclei can be assessed using confocal immunofluorescence after DAPI-staining [14, 20]. We found that treating H2542 MPM cells with 3 μM niraparib for 72 h generated threefold more micronuclei and twofold more bridging (Fig. 2c, supplemental Fig. S3). This was similar to the phenotype seen in other HR-deficient cancer cells [12, 14, 20].

### PARP1 inhibition results in increased sister chromatid exchange in MPM cells

Replication fork structural damage and subsequent repair often result in the exchange of DNA from one sister chromatid to another [17, 18]. Thus, sister chromatid exchange (SCE) correlates with the rate of damaged replication forks that have attempted repair, and all replication stressing agents induce SCE. We found that the incidence of SCEs is significantly increased when H2452 MPM cells are treated with 3 μM niraparib for 48 h and released into fresh media without the drug for 24 h. There was a ninefold increase in SCEs in cells treated with niraparib compared to the vehicle control group (Fig. 2d, supplemental Fig. S4). This demonstrated that the niraparib-exposed MPM cells endured far more damaged replication forks than untreated cells, consistent with the γ-H2Ax data.

## Discussion

MPM responds poorly to current chemotherapy or radiation therapy, and many presenting cases are unresectable due to the spread of the disease along pleural planes or chest wall invasion [1, 2]. The average survival of patients with recurrent or unresectable disease treated with the best chemotherapy regimen (cisplatin or carboplatin plus pemetrexed) is one year [1–3]. Adding the angiogenesis inhibitor bevacizumab to this regimen adds another 6 months of survival [1, 2]. However, all unresected or recurrent patients eventually die of their disease [1–3]. Many patients with unresectable MPM choose palliative care instead of systemic chemotherapy because current regimens are difficult to tolerate [2]. Targeted inhibitors against EGFR or VEGFR have been disappointing in their clinical benefit, so there are no approved precision therapies [2]. Thus, new therapies are needed for this devastating disease.

Between 23 and 64% of malignant pleural mesotheliomas (MPMs) have somatic inactivating mutation in the BAP1 gene [4–7]. Mutation in BAP1 leads to defects in the HR DNA repair pathway, and it is considered a tumor suppressor [6–8]. The HR pathway is required for faithful repair of stalled replication forks, and HR-deficient cells become genomically unstable [12–14]. This genomic instability can result in fusion of the stalled replication forks, generating chromosomal ligations [12–14, 20]. Thus, BAP1 deficiency may be an initiating event in oncogenesis because stalled replication forks are no longer faithfully repaired due to the lack of HR [12–14]. This is similar to the inherited HR deficiencies, such as the BRCA1 and 2 mutations, that lead to breast and ovarian cancers [12, 13].

PARP1 inhibitors are widely used as drugs that target these BRCA1 and 2-mutant cancers [12, 13, 18, 19]. PARP1 is essential for three DNA repair pathways—single strand-break repair, base excision repair (BER), and alternative non-homologous end joining (aNHEJ). Inhibition of PARP1 by the clinical PARP1 inhibitor drugs results in the synthetic lethal death of BRCA1 or 2 deficient cells. This cell death has multiple etiologies, all involving chromosomal instability and mitotic catastrophe [12–14, 20].

In the absence of PARP1 activity, SSBs increase in genomic DNA, either from defective SSB repair or arrested BER [13]. The unrepaired SSBs are converted into DS ends upon replication fork arrival [12]. In the absence of HR, replication fork collapse can be rescued by the aNHEJ repair pathway, but this pathway also requires PARP1 [14]. When PARP1 is inhibited in HR-deficient cells, there is an increased possibility that collapsed replication forks can be aberrantly ligated together with classical NHEJ. This leads to chromosome fusions and genomic instability, manifested by micronuclei, and bridging, as seen here [12, 20]. Another mechanism of replication fork stalling and collapse in these HR-deficient cells is that the clinical PARP1 inhibitors lock PARP1 onto DNA, blocking progression of a replication fork [13, 18]. In that mechanism, replication forks would also be arrested at the PARP1 inhibitor-DNA complex, and that cell would lack both HR and aNHEJ activities, and thus would have great difficulty in restarting that stalled fork [12, 13, 18].

Olaparib is an oral PARP1 inhibitor that has been recently approved for the treatment of relapsed ovarian cancer that harbors BRCA1 or 2 mutations [12, 13]. Niraparib is another approved potent oral PARP1 inhibitor that is well-tolerated [18, 19]. Results of a large clinical trial using single agent Niraparib in relapsed ovarian cancer demonstrated that it was a safe and highly effective agent in ovarian cancer, regardless of HR or BRCA1/2 status [19]. The data presented here demonstrate that PARP1 inhibition using niraparib or olaparib was lethal in multiple MPM cell lines, regardless of BAP1 mutational status. The finding that niraparib decreased replication fork restart (Fig. 2a) and increased genomic instability (Fig. 2c, d) is consistent with the molecular mechanism of synthetic lethality in PARP1 inhibition in BRCA1 and 2-deficient breast and ovarian cancers [12, 13, 18].

Bott et al. stated that they found no difference in the sensitivity to niraparib between BAP1-mutant and wild-type MPM using a cell respiration assay [7]. There was no data shown and there was no mention of whether niraparib was cytotoxic in any of the cell lines [7]. Using colony formation assays to measure cytotoxicity, which assesses not just the respiration of a cell population but clonal cell proliferative capacity, we also found that there was little difference in sensitivity to niraparib between BAP1-mutant or wild-type MPM cell lines. However, we found that niraparib was significantly cytotoxic to each MPM cell line, regardless of the BAP1 status (Fig. 1). This is consistent with a recent clinical trial where niraparib demonstrated efficacy in ovarian cancer regardless of HR status [19]. Olaparib also demonstrated cytotoxicity to all BAP1-mutant MPM cell lines (Fig. 1, supplemental Fig. 1). In H290 BAP1 wild-type MPM cells olaparib had significant cytotoxicity, while in the CRL-2081 BAP1 wild-type MPM cells it has less benefit (Fig. 1e, f, Supplemental Fig. 1).

There are two potential reasons why both wild type and mutant BAP1 were sensitive to niraparib. First, there could be undefined HR defects in the BAP1 wild-type MPM cells. It is possible that all MPM results from HR deficiencies, with BAP1 just being the most common type of deficiency [4–7]. Second, niraparib is such a potent PARP1 inhibitor that unrepaired oxidative DNA damage leads to such massive replication fork collapse that neither an HR-deficient or replete cell can tolerate it. These two mechanisms are not mutually exclusive, and niraparib could be lethal from inhibiting BER just as much from deficient SSB repair or aNHEJ [13, 14].

PARP1 inhibition at clinically relevant concentrations resulting in significant cytotoxicity in MPM demonstrates that agents such as olaparib and niraparib are promising for use in the treatment of MPM, for which effective treatment is desperately needed. Thus, clinical trials of PARP inhibitors in this difficult to treat malignancy would be warranted. These data are also a further demonstration that other malignancies with acquired defects in HR may be responsive to PARP1 inhibition [8]. Studying the effects of PARP1 inhibition in the other BAP1-mutant malignancies, such as renal cell carcinoma and cholangiocarcinoma, would be especially important, since cancers also have little beneficial therapy once initial resection has failed [8].

## Electronic supplementary material

Below is the link to the electronic supplementary material.

Supplementary material 1 (PPTX 1168 kb)
