# Supplementary material for: Synthetic lethality in malignant pleural mesothelioma with PARP1 inhibition
Source: Cancer Chemother Pharmacol. 2017 Jul 29;80(4):861–7. doi: 10.1007/s00280-017-3401-y (PMC5608777; doi:10.1007/s00280-017-3401-y)
Supplement: Supplementary file 1 — Supplementary material 1 (PPTX 1168 kb) [file 280_2017_3401_MOESM1_ESM.pptx]

## Slide 1
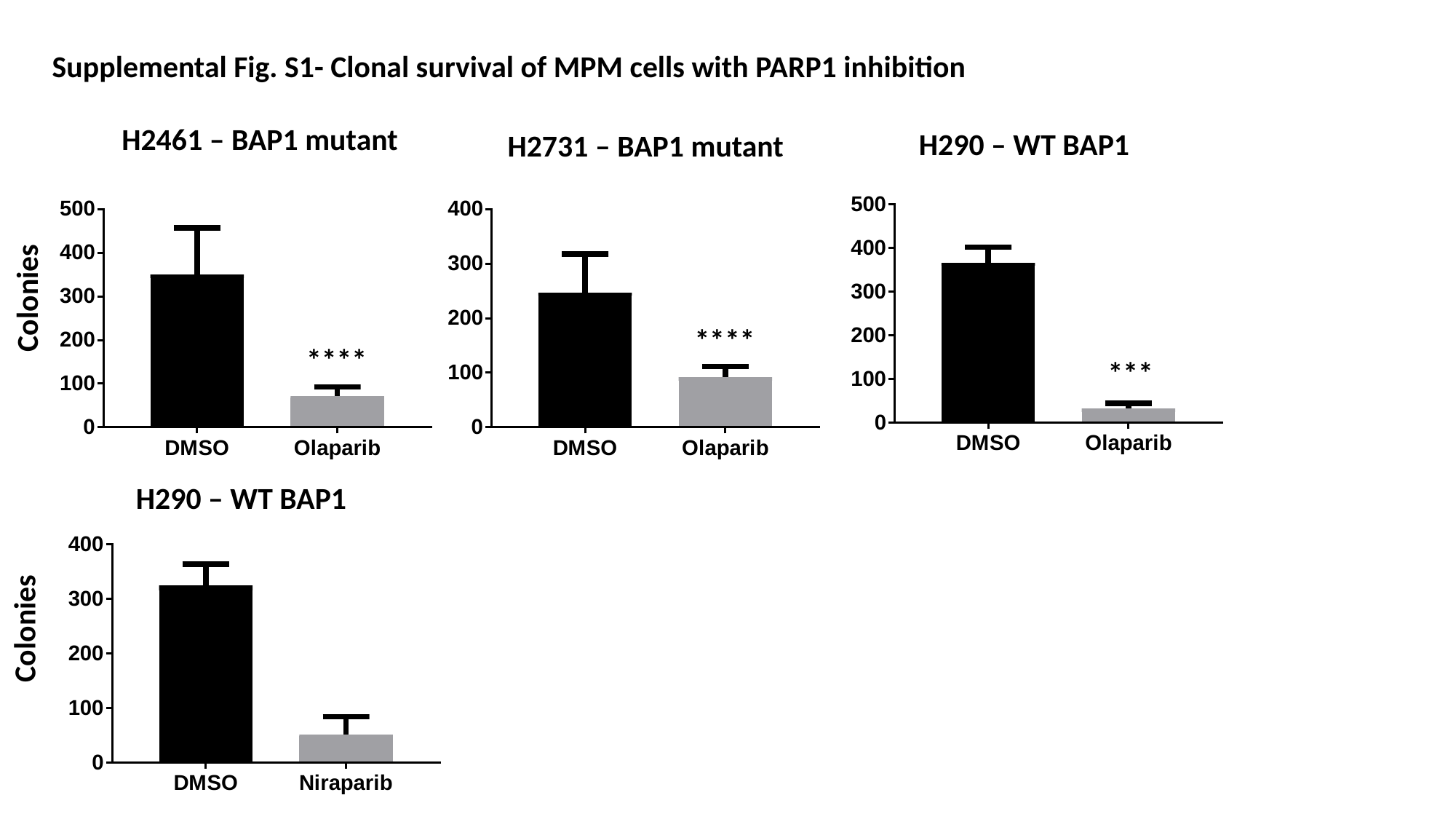

Supplemental Fig. S1- Clonal survival of MPM cells with PARP1 inhibition
H2461 – BAP1 mutant
H290 – WT BAP1
H2731 – BAP1 mutant
Colonies
****
****
***
H290 – WT BAP1
Colonies

## Slide 2
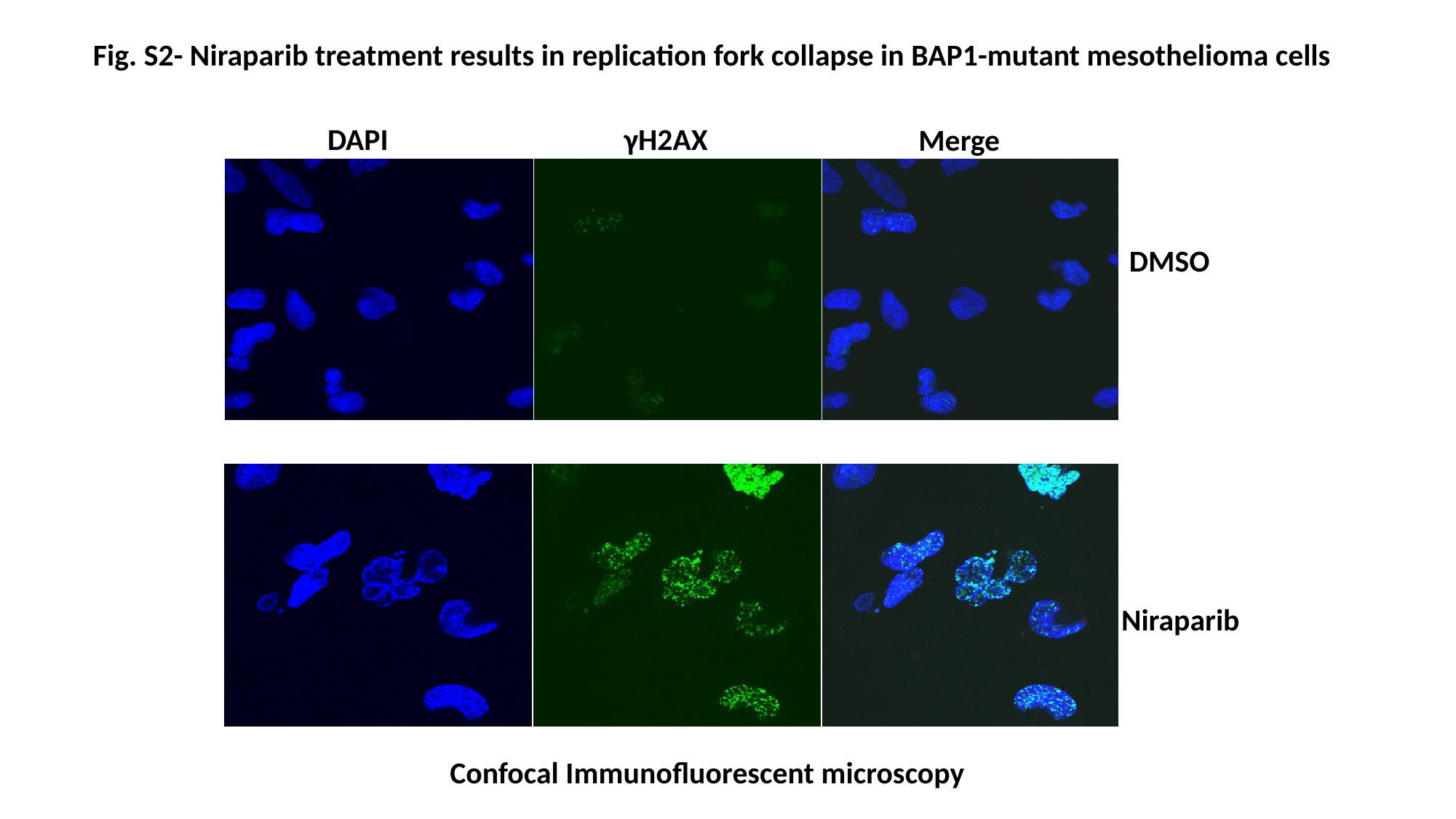

Fig. S2- Niraparib treatment results in replication fork collapse in BAP1-mutant mesothelioma cells
DAPI
γH2AX
Merge
DMSO
Niraparib
Confocal Immunofluorescent microscopy

## Slide 3
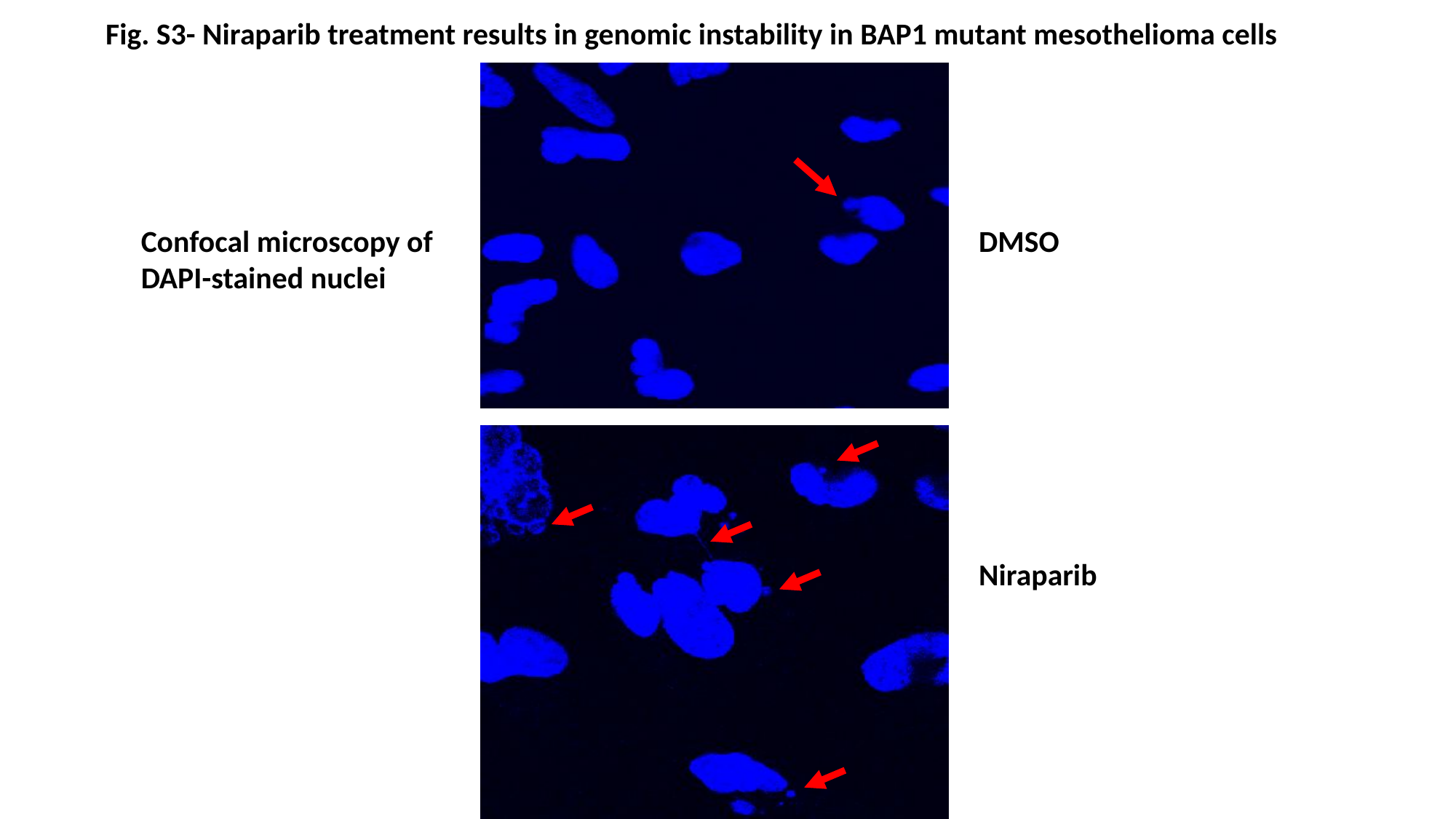

Fig. S3- Niraparib treatment results in genomic instability in BAP1 mutant mesothelioma cells
Confocal microscopy of DAPI-stained nuclei
DMSO
Niraparib

## Slide 4
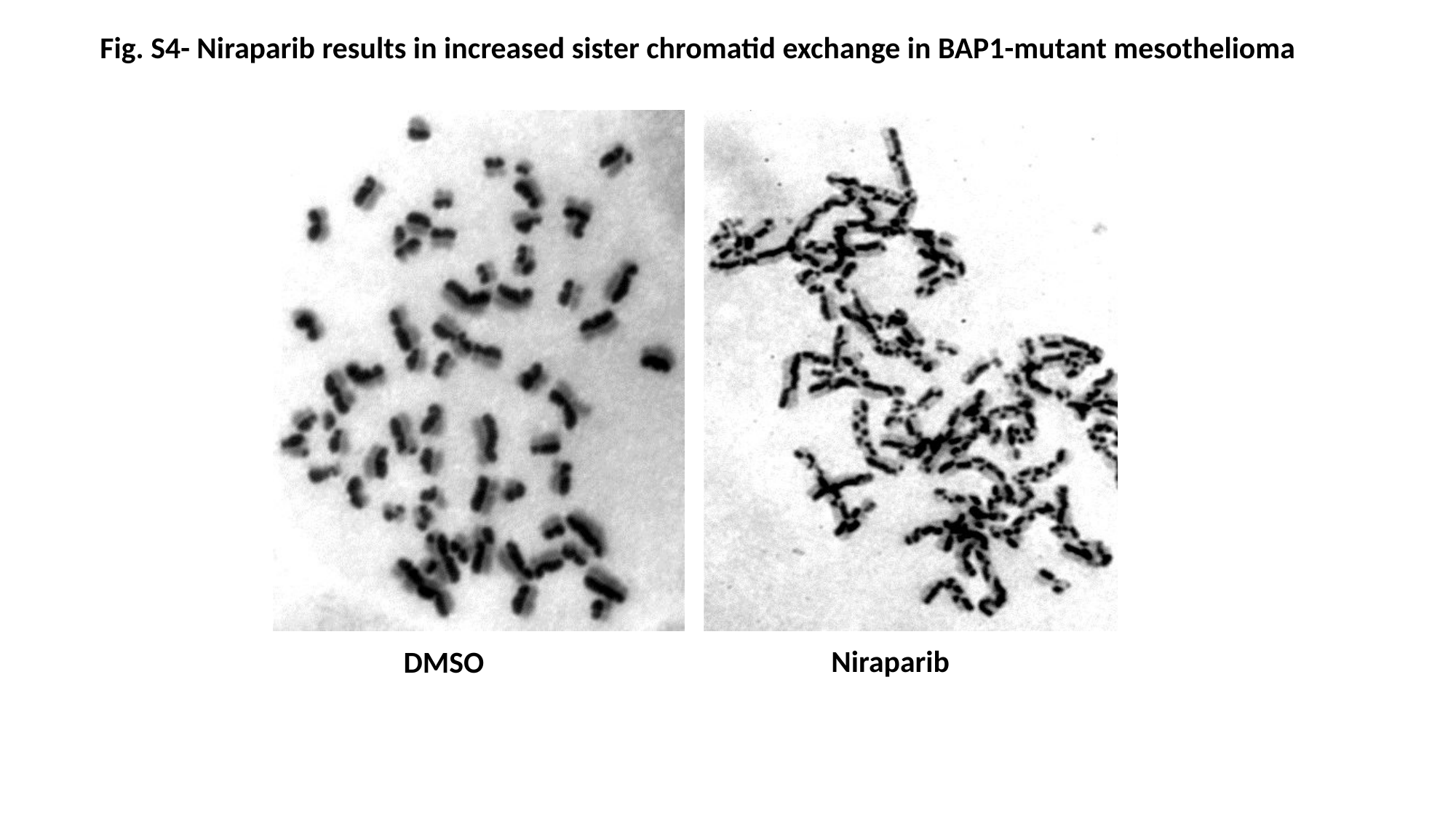

Fig. S4- Niraparib results in increased sister chromatid exchange in BAP1-mutant mesothelioma
Niraparib
DMSO
